# Mechanical Characteristics of Cement-Based Grouting Material in High-Geothermal Tunnel

**DOI:** 10.3390/ma13071572

**Published:** 2020-03-29

**Authors:** Mingnian Wang, Yunpeng Hu, Cheng Jiang, Yicheng Wang, Dagang Liu, Jianjun Tong

**Affiliations:** 1 Key Laboratory of Transportation Tunnel Engineering, Ministry of Education, Southwest Jiaotong University, Chengdu 610031, Chinatongjianjun213@163.com (J.T.); 2 School of Civil Engineering, Southwest Jiaotong University, Chengdu 610031, China

**Keywords:** high-geothermal tunnel, grouting material, varying temperature curing, mechanical strength

## Abstract

The cement-based grouting materials used for practical purposes in high-geothermal tunnels are inevitably affected by humidity and high temperature, leading to the deterioration of mechanical properties. Based on the characteristics of changing high temperatures and two typical conditions of hot-humid and hot-dry environments in high-geothermal tunnels, many mechanical strength tests were carried out on the grouting material cured under different environmental conditions. The study results indicated that high temperature and low relative humidity were unfavorable to the development of mechanical characteristics of grouting material, but the coupling effect of two factors could improve the strength at early ages and reduce the degradation of long-term strength. As the curing temperature exceeded 56.3 °C, the humidity effect on strength played a more important role in recovering the strength of grouting material damaged by high temperature. Temperature had more significant impact on the relative peak stress while the relative humidity had greater influence on the relative peak strain. A calculation compressive constitutive model was prospered, which considering both temperature and relative humidity. The study results may provide much valuable experimental data and theoretical supporting for the design of compression constitutive of cement-based grouting material in high-geothermal tunnel.

## 1. Introduction

Grouting has been widely applied in tunnels, subways, foundation pit of buildings and many other underground constructions [1]. The commonly used grouting materials in transportation tunnels are cement-based silicate engineering materials. As reported in [2], the flowing slurry can transfer to the inner cracks in surrounding rocks or artificially drill holes. After solidification and hardening of the grouting body, the integrity of the broken surrounding rock or the bonding properties between anchor rod and rock can be improved significantly, and thus increase the security of supporting structure. As one of the primary means of disaster prevention and control, the reinforcement effect of grouting has achieved good results in most tunnel constructions. However, with the development of transportation engineering, more and more mountain tunnels worldwide are being built under high-geothermal conditions. Especially in the Sichuan-Tibet railway project of China, dozens of transportation tunnels are facing the challenge of high-geothermal problem during the construction [3]. The rock temperature tested in the holes on site is almost always above 40 °C, which has a great influence on the reinforcement effect of grouting material, and thus poses a potential threat to the long-term safety of bolt-supported structure [4]. Therefore, deeper investigations on the mechanical characteristics of cement-based grouting material in high-geothermal tunnels are necessary.

In previous studies, scholars have conducted many experiments on the mechanical properties of grouting material under normal temperature conditions. Some studies have discussed grouting methods, various grouting parameters, and the properties of grouting material appropriate for tunnel construction [5]. Generally, there are many factors that have a significant impact on the mechanical behavior of cement-based grouting material, such as w/c ratio, curing condition, substance components, specific surface area, viscosity and so on [6]. Li et al. [7] conducted many experiments on modified additives for grouting material and proved that higher water to binder ratio led to good fluidity, but the compressive strength decreased significantly. Li et al. [8] studied a new cementitious anti-washout grouting material (CIS). The results indicated that the CIS grout had a high early compressive strength due to the admixture of water glass. The hydration products of C-S-H cause CIS grout to be denser, lending it a higher strength, but the increase of xanthan gum has an opposite effect. Some investigations have indicated that the early strength of most cement-based grouting materials can be improved by mixing lithium carbonate hardening accelerators, but the test results by Won et al. [9] suggested that this additive may also lead to a decrease in long-term strength. However, the focus of those investigations was mainly on the characterization of cement grouts at standard room temperature.

As reported in [10], the real construction environment, particularly as presented recently for high-geothermal tunnels, has a temperature of about 40 °C to 90 °C. The impact of high temperature on the mechanical properties of grouting material has to be fully taken into consideration. On the one hand, several studies on the temperature effect (<40 °C) have indicated that the hydration degree of the grouting material, which determines the hardening, is known to depend largely on temperature [11]. Consequently, in [12], Mirza et al. found that the setting time of grouting material significantly depended upon the temperature variation, and the increase of temperature can accelerate the condensation of most kinds of grouting material. In addition, curing temperature has a remarkable influence on the strength properties of cement grout specimens. Elkhadiri et al. [13] studied cement pastes specimens cured under higher temperature (e.g., at 22 °C and 40 °C), finding that they had a higher strength than those cured under lower temperature (e.g., 4 °C). Holt et al. [14] proved that early strength is vital to supporting tunneling, as the cement-based grouting material used in tunneling engineering and the lower curing temperature may obstruct the development of compressive strength in the early stages, resulting in the invalidation of rock bolts or the failure of the supporting system. Bohloli et al. [15] also found that the rapid increase in temperature in grouting material had a negative effect on the strength of grout specimen. The experimental results indicated that a high temperature of 20 °C led to a lower uniaxial compressive strength of the grouting body than that found at 8 °C. Although those findings made great contributions to the study on the impact of temperature on grouting material properties, more and more high-geothermal tunnel project cases are presenting rock temperatures far beyond the previously investigated temperature range. On the other hand, many scholars have also carried out research on grouting materials that have experienced fires [16]. Wang et al. [17] found that under temperature conditions of 550 °C, the ultimate bearing capacities of cement-based material samples cured at 14 and 28 days decreased to 41% and 60%, respectively. Li et al. [18] studied the residual compressive strength of cement-based grouting material by exposing the samples to the temperatures of 150 °C, 350 °C, 550 °C, and the testing results indicated that being exposed to higher temperature results in lower mechanical behaviors. Similar results were obtained in the investigation on the elastic modulus of grouting material with early age after fire [19]. However, the cement-based grouting materials in those researches had been hardened sufficiently before being exposed to the fire condition, and there exist essential differences between the two kinds of high-temperature environments when compared to the grouting material used in high-geothermal tunnels.

Additionally, on the basis of our previous study results [20,21], various cooling measures have been taken in the engineering of high-geothermal tunnels in order to create an appropriate working environment. Therefore, the grouting material used in actual high-geothermal tunnels is hydrated and solidified under variable temperature curing (VTC) conditions instead of under constant high-temperature curing conditions [10]. In addition to the problem of high temperature, the influence of relative humidity (RH) in high-geothermal tunnels on grouting materials should not be underestimated. Some studies have suggested that it is easy to cause decreases in compressive strength and bond strength in grouting material in a hot-dry environment [22,23]. There exists a significant combined effect of temperature and relative humidity. In summary, the real environmental conditions in high-geothermal tunnels is far complex, and current studies considering the environmental effect (temperature and humidity) on the mechanical properties of grouting material are limited. Thus, the major aims of this paper are:

a. Perform a series of experiments on the compressive strength of grouting material cured under VTC conditions (all combinations of temperature and RH).

b. Study the failure characteristics and mechanism of grouting materials.

c. Discuss the impact of temperature and RH, including the coupling effect on the mechanical properties of grouting material, and establish a compressive strength prediction formula.

d. Obtain a constitutive model of common cement-based grouting material subject to high-geothermal environments.

The results of this investigation may provide valuable information and theoretical support for grouting technology, numerical models of grouting materials, and the supporting design in high-geothermal tunnel.

## 2. Experiment Design

### 2.1. Raw Materials and Specimen Preparation

The mix proportion of grouting material was determined with reference to one typical high-geothermal tunnel in Lhasa-Nyingchi railway, China. According to the Portland cement standard [24], ordinary type 1 Portland cement (produced by southwest cement factory of China) was selected as the binder in the tests, whose strength grade was 42.5 MPa. The cement density was 3.0 g/cm^3^. The initial setting time was 115 min and the final setting time 185 min. As a fine aggregate of the grouting material, the diameter of the selected sand was in the range of 0.5–0.35 mm, and the fineness modulus was 2.12. The mixing ratio was 0.5:1:1 (water:cement:sand), and no other admixtures were used in the testing samples. The particle size distribution of cement and sand are shown in Table 1 and Table 2. The kind of cement mortar used in the experiments was M35, and the chemical compositions are shown in Table 3.

**Figure 1:**
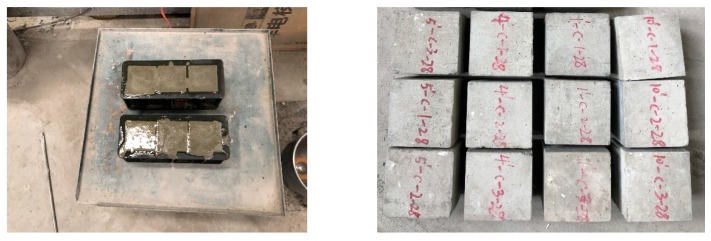
Specimen preparation of cement-based grouting material.

The testing samples for uniaxial compressive strength were formed into cubes whose size was 70.7 mm × 70.7 mm × 70.7 mm. The mixtures were filled into the testing mold simultaneously. The vibration of the grouting material should be done with a plate-type vibrator associated with an insertion-type vibrator, so as to ensure that the cement mortar is filled with the test mold in a compact manner, avoiding the stratification and segregation of the cement mortar. It was necessary to scrape the excess cement mortar on the test mold and smooth the surface with spatula (see Figure 1). After the initial setting of the cement mortar, the specimens were put into the appropriate apparatus for curing.

### 2.2. Experimental Procedures

#### 2.2.1. Curing Method for Samples

**Figure 2:**
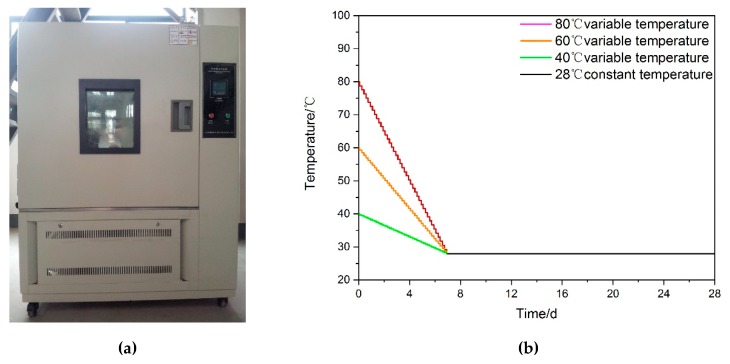
Curing device and temperature curing curve under VTC condition. (**a**) curing box, (**b**) curing temperature curve.

Based on our preliminary experimental work, the testing specimens were cured under VTC conditions by HX/HS-010L curing apparatus (produced by Huixia instrument factory in Shanghai, China) in Figure 2a [10,21]. The precision of temperature and relative humidity regulation reached 0.1 °C and 0.1%, respectively. To simulate the real environmental conditions in high-geothermal tunnels, the curing temperature was set at a fixed value after putting the samples into the curing box of the device. In the following curing process, the temperature declined at the same rate every 8 h, and the change of temperature occurred three times a day. As shown in Figure 2b, when the temperature was gradually reduced to 28 °C over 7 days, the automatic temperature control device remained stable until 28 days. The relative humidity in the curing box was set at a constant value from beginning to end. All changes in environmental factors were performed by a servo control system of the curing apparatus. According to our previous field test results and investigations on some typical high-geothermal tunnels, the grouting material specimens were tested at different combination of temperature (40 °C, 60 °C, 80 °C) and relative humidity (25%, 55%, 95%) [25,26,27]. Moreover, the compressive strength of grouting specimens cured at 3 days and 28 days were selected as the main study subjects. Meanwhile, as a comparison, another group of testing samples cured under standard curing (SC) conditions was produced. The curing temperature and relative humidity were maintained at 20 °C and 95%, respectively. To improve the test accuracy, the number of effective specimens should not be less than three under each test condition and the average strength was defined as the effective results. There were 64 testing samples in total in this experiment (including four invalid specimens). The test details are presented in Table 4.

#### 2.2.2. Uniaxial Compression Test

**Figure 3:**
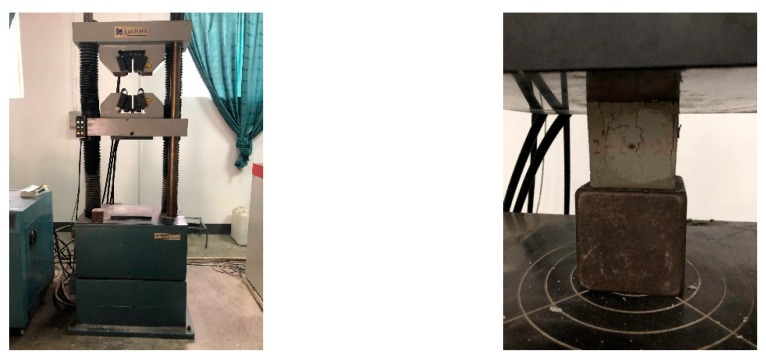
Equipment for uniaxial compression test.

The mechanical strength tests on grouting material specimens were conducted by CSS-WAW600D electric-fluid servo compression machine (produced by the testing machine research institute in Changchun, China). The maximum test force of the machine was 600 kN and the moving speed of piston can be set below 100 mm/min. The accuracy of displacement and velocity were able to reach ±0.5%. Moreover, as shown in Figure 3, stiff inserters with a height-to-diameter ratio of 1.0 were inserted between the specimens and the platens in order to make the stress distribution in the specimen much more uniform. The specimen was preloaded to reduce the impact of the uneven surface and the gap between the compression surface and loading surface. A hydraulic servo press system was adopted to load continuously and evenly. In order to obtain the full stress-strain curve, the strain controlling mode was selected to ensure a constant strain rate at all stages during the whole testing process, and the loading speed was 400–600 micro strain/min. The oil pump and electro-hydraulic servo valve were controlled by PC servo controller for loading and unloading, which is able to realize multi-channel closed-loop control, and thus completely automatic control loading, and automatic measurement and data collection during the test process. All samples were tested in accordance with the method set out in GB50081-2002. The test results for the relationships among temperature, relative humidity and stress-strain are shown in Table 5.

## 3. Results and Discussion

### 3.1. The Coupling Effect of Temperature and RH on Compressive Strength

**Figure 4:**
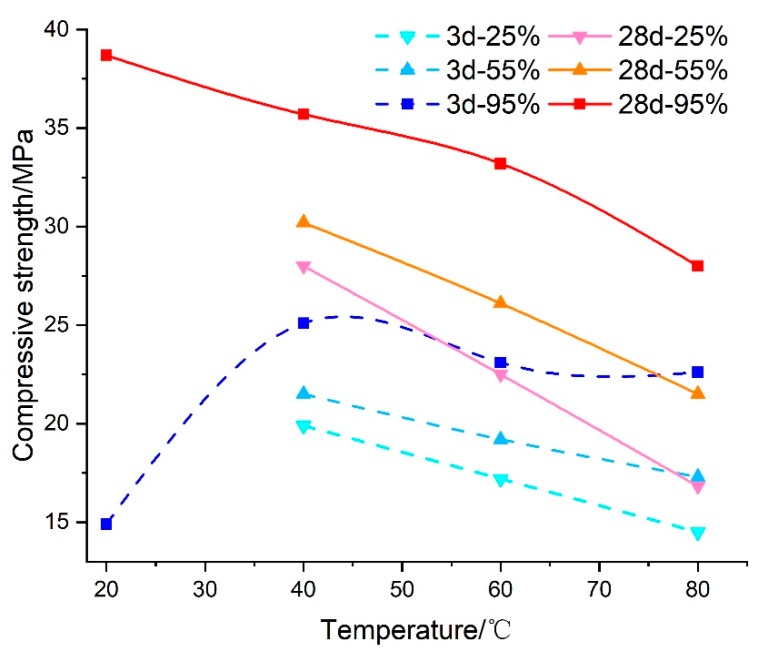
The impact of temperature on the compressive strength of grouting material.

The testing results of samples cured under different curing conditions indicated that compressive strength was influenced significantly by temperature and relative humidity, but their effects and mechanism of influence on the mechanical properties were quite different. As shown in Figure 4, there was a negative correlation between the compressive strength and curing temperature. Whether the curing age was at 3 d or 28 d, the compressive strength decreased with the increase of temperature, and the higher the curing temperature was, the larger the decline in mechanical strength was. When compared to curing at 40 °C, the compressive strength for other testing samples cured under higher temperature conditions fell, with drops of between 10% and 40%. Of all curing temperature conditions, 80 °C–3 d–25% produced the lowest compressive strength and the strength decline under 80 °C–28 d–25% was the greatest of all conditions. The main reason was that high temperature may speed up the hydration of cement in grouting material in an environment with sufficient free water. However, the strength development of grouting material cured under constantly high temperature could be inhibited by lower relative humidity, and the longer the curing time lasted, the more significant the strength reduction would be. This means that temperature is one of the most influential factors on compressive stress, but not the only affecting factor.

**Figure 5:**
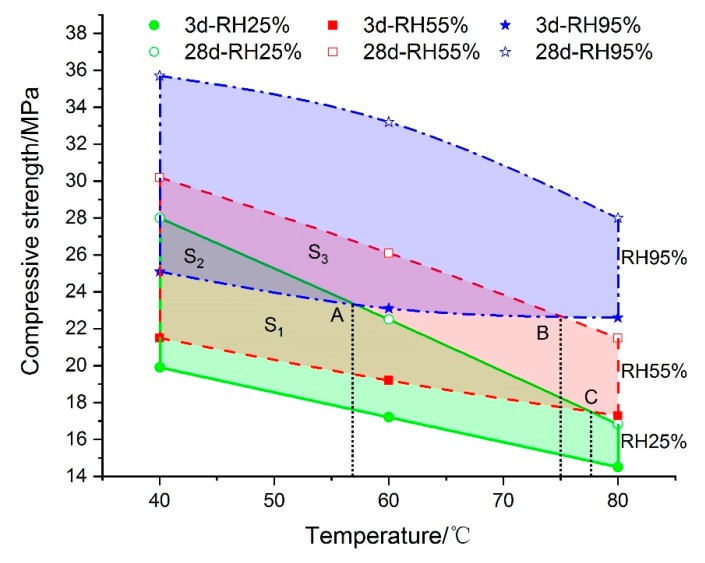
The impact of RH on the compressive strength of grouting material.

The impact of relative humidity on compressive strength can be seen in Figure 5. It indicates that the rise in relative humidity contributed greatly to the increase in compressive strength, regardless of curing time. When the curing age was set at 3 d, the minimum increase of compressive strength reached by 26.1% due to the rise of RH from 25% to 95%, while it was 27.5% when the setting time was 28 days. However, the gain effect on mechanical properties by relative humidity was more significant with the rise of temperature. When the curing temperature reached 80 °C, the biggest increase of compressive strength was over 52% compared to 40 °C, and the growth rate obviously accelerated as temperature rose. This can also be seen from the overlapping area of S_1_, S_2_ and S_3._ When the difference of curing humidity was large (like 25% and 95%), the compressive strength of testing samples cured at 28 d was smaller than that at 3 d, which meant that the curing age had little effect on the development of mechanical performance when the temperature exceeded 56.3 °C. Similar results can be found in the condition of RH55% and RH95% when the curing temperature was over 75 °C and 77.5 °C. It can be concluded that the increase in relative humidity is beneficial for the development of compressive strength, and the RH effect can further intensify under higher curing temperature.

**Figure 6:**
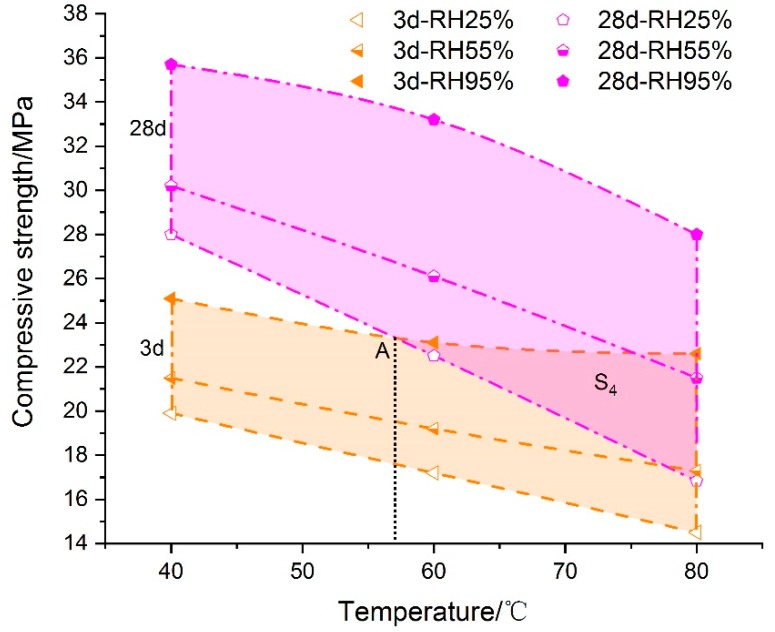
The impact of curing age on the compressive strength of grouting material.

Generally, the curing time had a positive effect on the development of mechanical strength of cement-based grouting material. As shown in Figure 6, the compressive stress of specimen cured at 3 d–95% was always lower than that at 28 d–25%, which meant that curing age played a major role in increasing the mechanical properties when the temperature was below 56.3 °C. Even though there was a 70% difference in relative humidity, the 28-day compressive strength at 40 °C–RH25% was 11.6% more than the 3-day compressive strength at 40 °C–RH95%. However, with the increase of curing temperature, the above situation changed gradually. As the overlapping area S_4_ became larger and larger, the coupling effect of temperature and humidity markedly improved the strength of the grouting materials at early ages. The difference of compressive strength caused by curing age decreased gradually. For example, when the temperature was set at 80 °C, the compressive strength of samples cured under 3 d–RH95% increased by as much as 18.6% and 4.9% compared to 28 d–RH25% and 28 d–RH55%, respectively. The temperature and relative humidity began to play a main role in improving the mechanical properties of grouting material.

### 3.2. Stress-Strain Characteristics in Compression

#### 3.2.1. Fracture Failure Process

**Figure 7:**
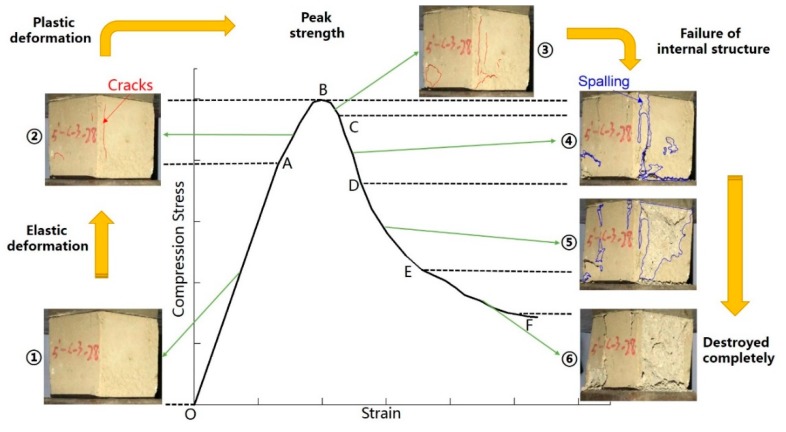
Compression failure process and characteristics of stress-strain curves of grouting material sample.

In actual high-geothermal tunnel constructions, the long-term strength of the composite supporting structure that consists of grouting material is one of the most concerning problems. Therefore, the testing samples cured at 28 days were selected as the main study object in the following stress-strain research. As shown in Figure 7, the general graph of stress-strain curves was obtained on the basis of multiple sets of compressive tests. The overall law of the curve change was basically the same, and similar results can be found in existing studies [27,28]. The development of curves show that the compression stress first rose rapidly to the peak value, then turning into a falling section and decaying thus to the residual strength. The OA segment was the linear elasticity increasing stage. First, the interface is contacted and the gap closes gradually. The compression stress increases linearly with the vertical strain. The slope of the straight line remains basically invariable; the compression stress in the AB segment grows nonlinearly with the increase of compressive strain, and the slope of the curve begins to decrease. Before reaching peak strength, the internal micro-crack gradually expands with a slight cracking sound, but only a few micro-fractures appear on the surface of the specimens. The stress-strain curve begins to decline in the BC segment. More and more short, thin vertical cracks that are not interconnected appear on the specimens’ surface. The rapid decline in the CD segment indicates that some local structures inside the samples have been damaged. The rate of vertical deformation increases due to the inadequate bearing capacity of samples. With the continued increase of normal stress, the decrease of stress slows down, accompanied by an increasing number of cement mortar spalling off, and finally the compressive stress gradually becomes equal to the residual stress.

**Figure 8:**
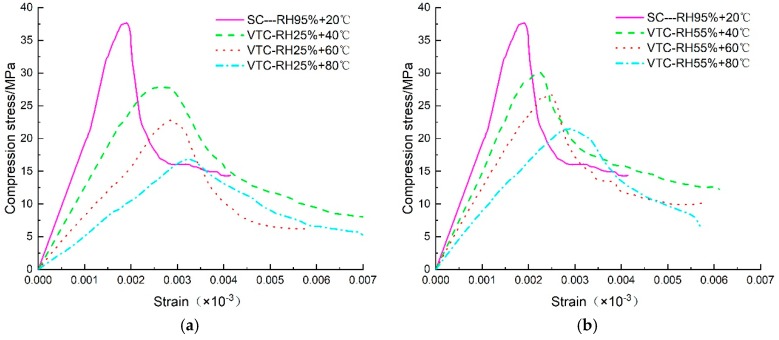

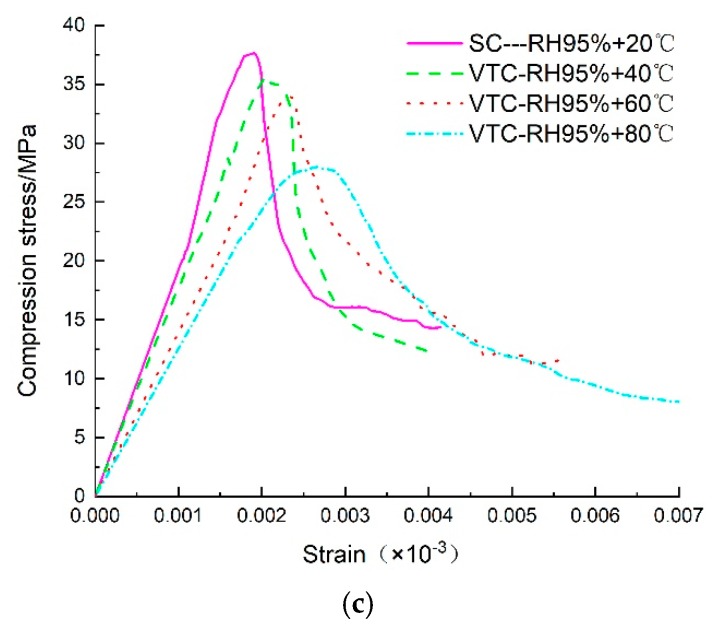
Compressive stress-strain curves under different curing conditions. (**a**) SC vs. VTC-RH25%, (**b**) SC vs. VTC-RH55%, (**c**) SC vs. VTC- RH95%.

However, the shape of the compression stress-strain curves was different due to the influence of curing temperature and relative humidity. With the increase of temperature, all testing samples showed a decrease in compressive stress. Compared to specimens cured under SC conditions, the compression stress-strain curve showed a lateral expansion with increasing temperature. The vertical change of the curve was from high to low, and the lateral change was from narrow to wide. This means that as the initial curing temperature increased, the drop of post-peak stress gradually slowed down, meaning a decrease in the degree of brittleness. However, the increase of relative humidity had the opposite effect on the development rule of compression stress- strain curves (see Figure 8).

#### 3.2.2. Peak Stress and Strain

**Figure 9:**
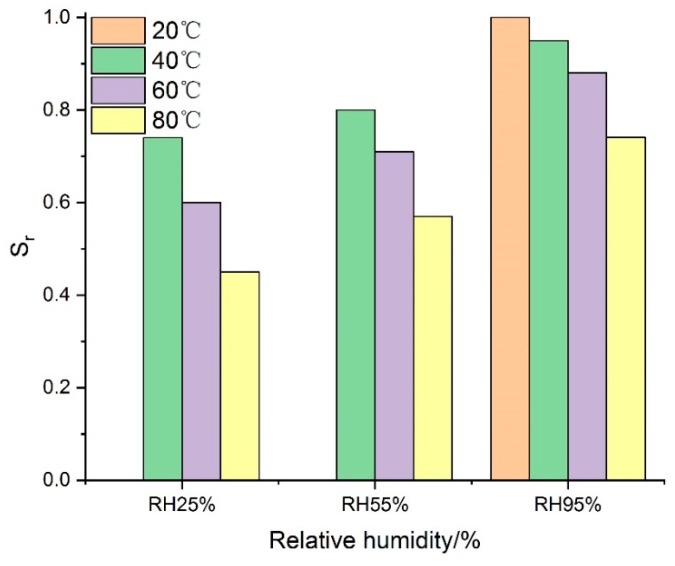
The influence of temperature and RH on S_r_.

To further investigate the strength degradation mechanism of grouting material under different temperature and RH, the maximum compressive strength of samples cured under VTC conditions divided by the peak strength of specimens cured under SC conditions was assessed as the relative peak stress (S_r_) of cement-based grouting material.

(1) $$S_{r}=\frac{\sigma_{max}^{VTC}}{\sigma_{max}^{SC}}$$

where $\sigma_{max}^{VTC}$ is the peak stress of specimens cured under VTC conditions, and $\sigma_{max}^{SC}$ is the peak stress of samples cured under SC conditions. As shown in Figure 9, S_r_ was always less than 1.0 under all VTC conditions, which means that the mechanical strength of grouting material in high-geothermal environments decreased to different degrees compared to normal conditions. That is why some engineering problems like failures in advanced pipe grouting or anchorage systems usually occur a long time after construction in high-geothermal tunnels [28,29,30].

**Figure 10:**
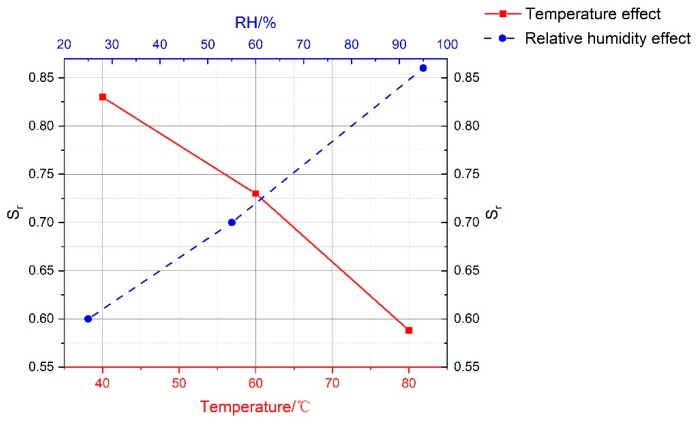
Relationship curves of average relative peak stress of testing specimens.

According to the relative peak strength, values of S_r_ under the conditions of different curing factors were obtained, and the higher the temperature, the lower the relative humidity, the more the compressive strength of grouting material declined. The testing results indicated that S_r_ at RH25% fell by as much as 39.9% when the temperature was increased from 40 °C to 80 °C, while it only fell by a maximum of 21.6% at RH95%. This means that there was a significant positive correlation between S_r_ and RH. Moreover, the greater the difference in S_r_, the more significant the effect on the mechanical performance of cement-based grouting material will be for this curing factor. Based on the simple effect results shown in Figure 10, the maximum difference values of S_r_ under different curing conditions can be obtained: the curing temperature was 29% and the curing RH was 31%. Thus, the RH effect was slightly greater than the temperature effect.

**Figure 11:**
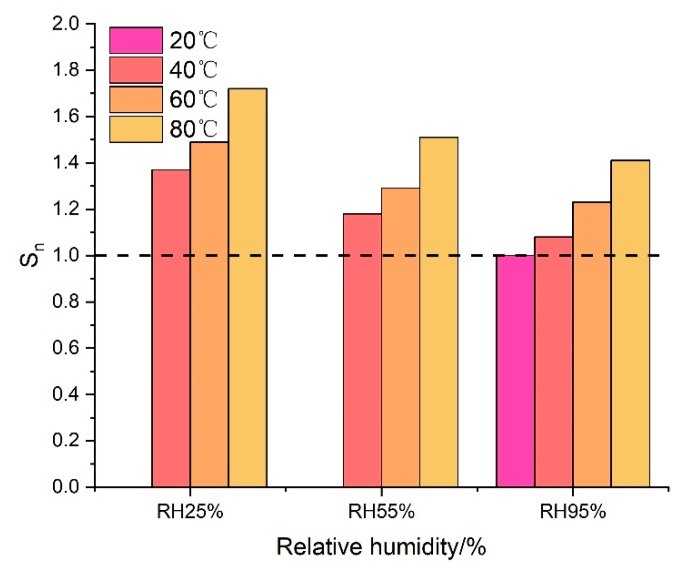
The influence of temperature and RH on Sn.

**Figure 12:**
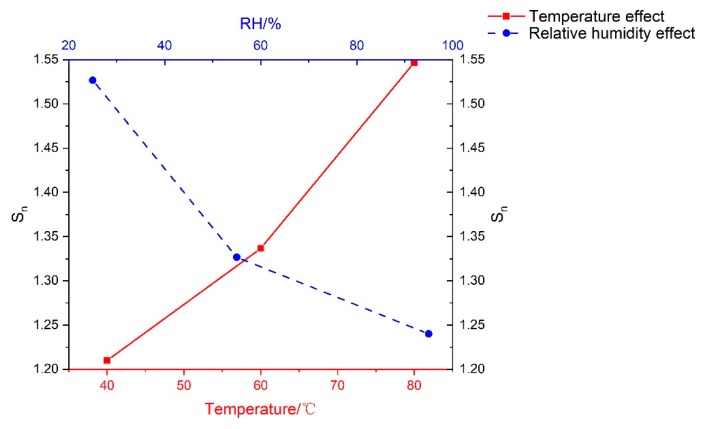
Relationship curves of average relative peak strain of testing specimens.

However, the conditions of high-geothermal environments have different effects on peak strain of grouting material. Similarly, the maximum compressive strain of samples cured under VTC conditions divided by the peak strain of specimens cured under SC conditions was defined as the relative peak strain (S_n_) of cement-based grouting material.

(2) $$S_{n}=\frac{\varepsilon_{max}^{VTC}}{\varepsilon_{max}^{SC}}$$

where $\varepsilon_{max}^{VTC}$ is the peak strain of grouting materials cured under VTC conditions, $\varepsilon_{max}^{SC}$ is the peak strain of grouting materials cured under SC conditions. As seen in Figure 11, S_n_ ranged from 1.08 to 1.72 in all VTC conditions, which meant that the peak strain of all testing samples increased in various degrees compared to that cured under SC condition. The higher the temperature and the lower the relative humidity were, the larger the S_n_ was. When the curing relative humidity was set at 25%, the value of S_n_ increased by 25% due to the rise of temperature, while it was 27.9% and 31% at RH55% and RH95%, respectively. Moreover, it was apparent that with the increase of RH from 25% to 95%, a temperature of 40 °C produced larger decrease of strain (22%) than 60 °C (17.5%) and 80 °C (18%). This indicated that S_n_ increased linearly with the curing temperature under different levels of RH. However, the RH effect on the decreasing of S_n_ was significant only under lower temperature. The maximum difference values of S_n_ under different curing conditions are shown in Figure 12, the temperature was 23.1% and the curing RH was 27.8%. Therefore, the temperature had a more significant influence on the peak strain of grouting material in high-geothermal environments than relative humidity.

#### 3.2.3. Quantitative Relationship between Curing Conditions and Relative Stress-Strain

**Figure 13:**
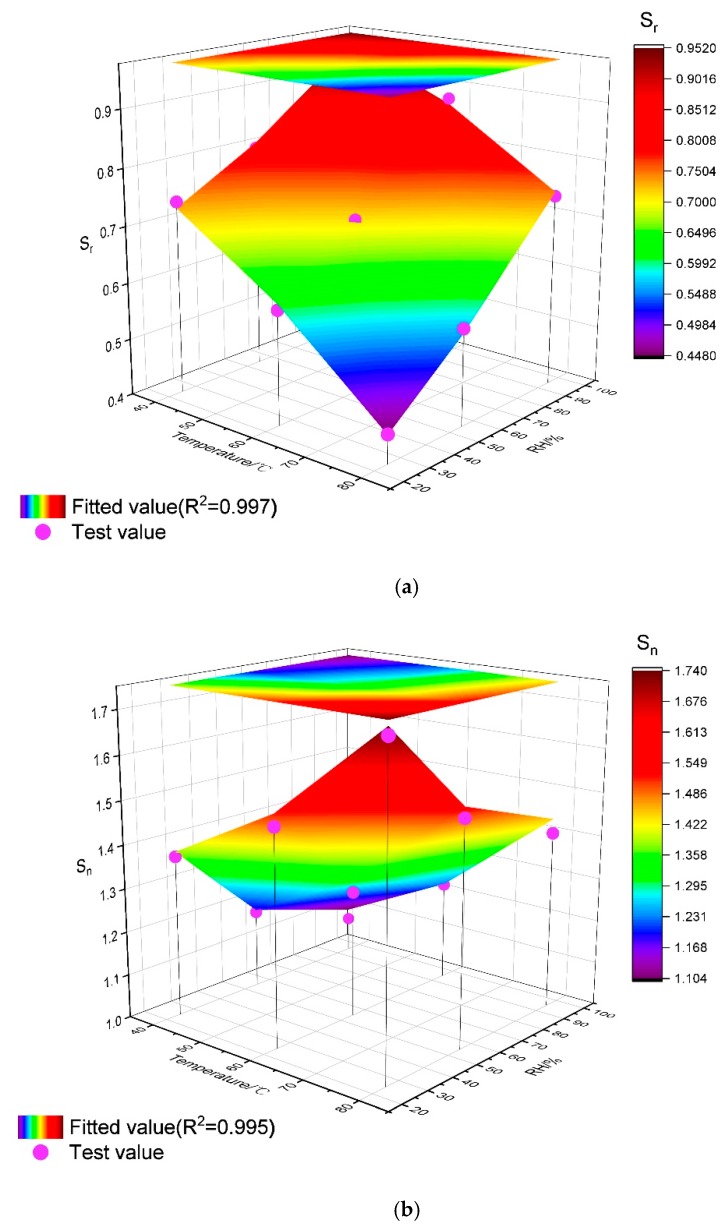
Multiple regression surfaces with projection of Sr and Sn. (**a**) Relationship between Sr and curing condition, (**b**) Relationship between S_n_ and curing condition.

To further study the interactions between typical curing conditions (temperature and RH) in high-geothermal environments and long-term compressive stress and strain of cement-based grouting material, the method of multi variable nonlinear regression analysis was selected. Temperature (T) and humidity (H) are considered as independent variables, S_r_ and S_n_ are taken as the dependent variables, and the relationship is defined by means of the following equation:

(3) $$S=a_{1}T^{2}+a_{2}H^{2}+a_{3}TH+a_{4}T+a_{5}H+a_{6}$$

where S is the peak parameter; T is the curing temperature; H is the curing relative humidity and a_i_ (I = 1–6) is the regression coefficient. According to the formula, the relationship between peak parameters (S_r_ and S_n_) and the conditions of high-geothermal environment can be obtained as shown in Figure 13, and the mathematical expression of S_r_ and S_n_ are presented as the following formulas, respectively:

(4) $$S_{r}=-5.42\times 10^{-5}T^{2}+1.23\times 10^{-5}H^{2}+2.77\times 10^{-5}TH-1.20\times 10^{-3}T+5.76\times 10^{-4}H+0.814$$

(5) $$S_{n}=1.04\times 10^{-4}T^{2}+6.43\times 10^{-5}H^{2}-6.76\times 10^{-6}TH-3.70\times 10^{-3}T-1.14\times 10^{-2}H+1.601$$

As seen in the surface with projection, the fitting coefficient R-squared of the quadric multiple regression equation of S_r_ is 0.997, while it is 0.995 for S_n_, which means a better correspondence of testing results. Thus, the mathematical equation is a quantitative description of the interaction between the conditions in high-geothermal environment and mechanical strength of grouting material.

### 3.3. Establishment of Constitutive Model

**Figure 14:**
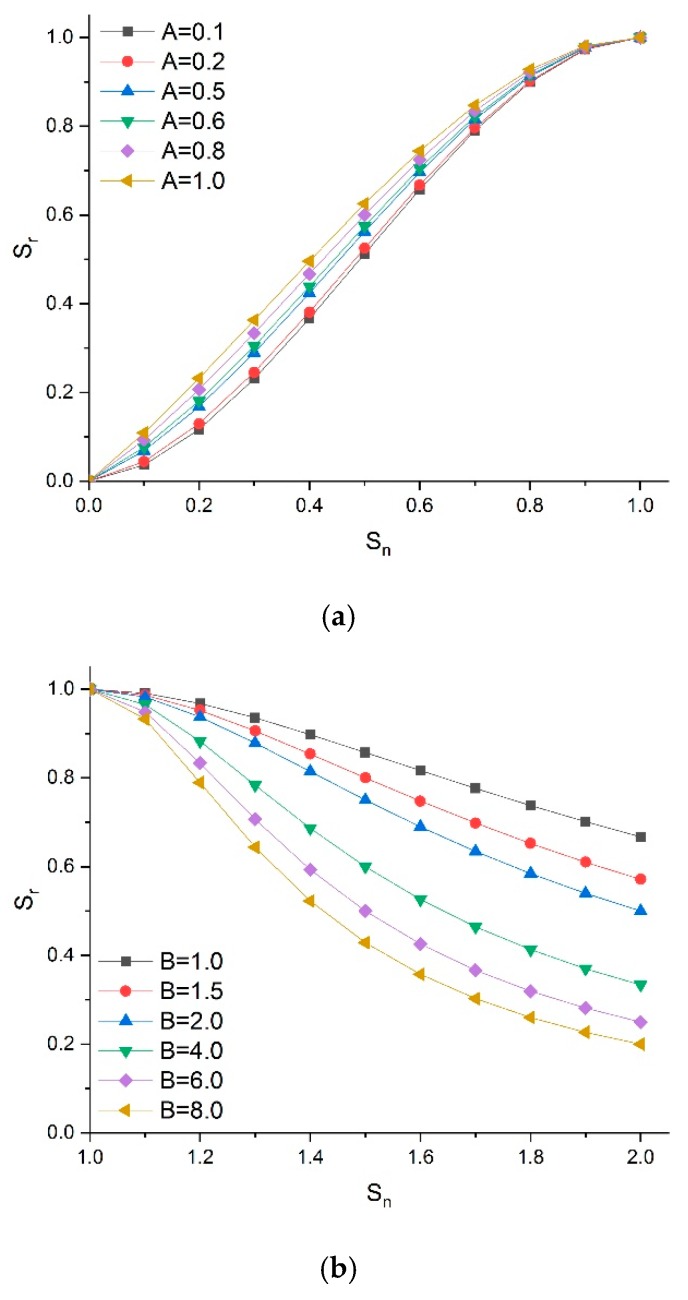
Characteristics of model parameters A and B. (**a**) Influence of parameter A on relative stress-strain relationship, (**b**) Influence of parameter B on relative stress-strain relationship.

It can be seen from the dimensionless processing of stress-strain that the compressive stress-strain curve of cement-based grouting material cured under VTC conditions has similar geometric characteristics to that under SC conditions. Through the research on the relationship between stress-strain and two influencing factors of high-geothermal environment, a new constitutive model of grouting material compressive strength in high-geothermal environment is obtained. Taking the dimensionless parameter S_n_ as the X-axis, and S_r_ as the Y-axis, the basic form of the upward part of the curve is described by the cubic polynomial, and the decline segment is presented as follows [31,32]:

(6) $$y=\mathrm{Ax}+\left(3-2A\right)x^{2}+\left(A-2\right)x^{3}$$

(7) $$y=\frac{x}{B{\left(x-1\right)}^{2}+x}$$

where parameter A reflects the change in the deformation modulus of the specimen under compression, which is equal to the ratio of the initial tangent modulus of the curve to the secant modulus at the stress peak of the curve. As shown in Figure 14, the smaller the parameter A, the greater the slope of the curve. The plastic deformation of grouting material was small with obvious characteristic of brittle failure. Parameter B reflects the area wrapped in the descending section of the curve. The smaller the parameter B, the larger the area under the stress-strain curve. The deformation of the specimen is large with obvious characteristic of ductility damage.

According to the testing results of specimens cured under SC and VTC conditions, the estimated parameters of the fitting formula and calculation model data are presented in Table 6. It can be seen that the smallest fitting coefficient R-square of the equation was over 0.9, which means that the two formulas can better express the stress-strain curves of grouting material under high-geothermal environments.

However, for convenient application of the constitutive model in practical engineering, it was necessary to further study the relationship between parameters (A, B) and curing conditions (temperature and RH). The fitting results in Table 6 indicate that parameters A and B generally decrease with the increase in curing temperature and increase with the rise in curing humidity. Therefore, the relationship between temperature, RH and the fitting parameters in the ascending and descending sections can be obtained as follows after further statistical regression analysis:

(8) $$A=0.89+3.69\times 10^{-3}H-5.38\times 10^{3}T-4.83\times 10^{-5}H^{2}+1.07\times 10^{-4}HT-6.89\times 10^{-5}T^{2}\;R^{2}=0.977$$

(9) $$B=2.32+1.97\times 10^{-3}H-5.60\times 10^{3}T+1.15\times 10^{-4}H^{2}-1.00\times 10^{-4}HT-5.27\times 10^{-5}T^{2}\;R^{2}=0.962$$

**Figure 15:**
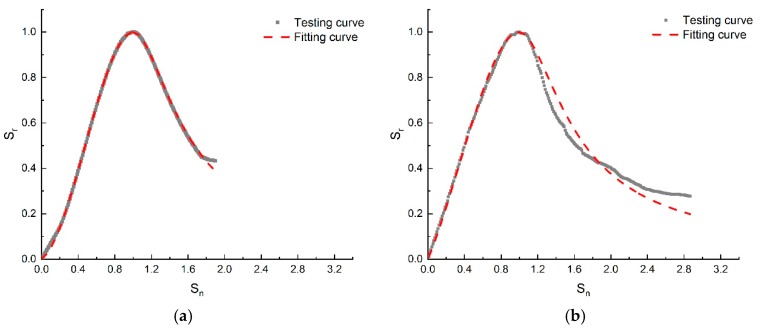

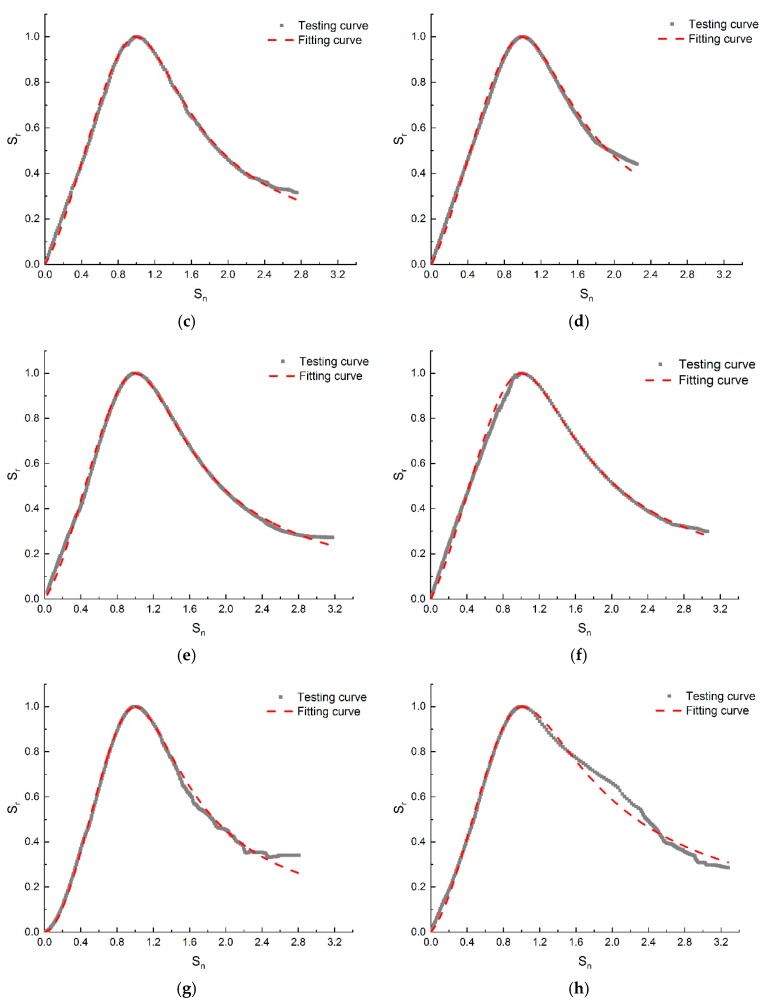

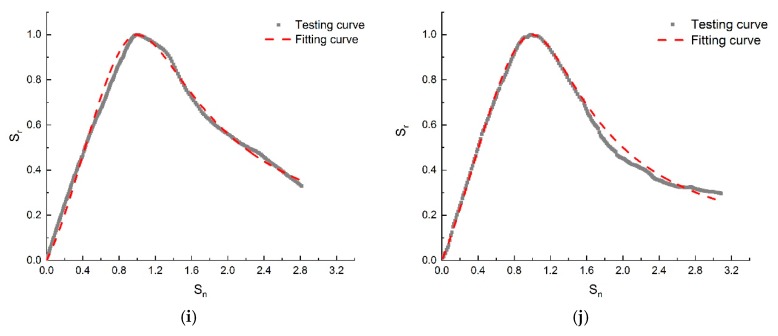
Comparison between the testing and fitting curves of specimens under different conditions. (**a**) 20 °C-RH95%, (**b**) 40 °C-RH25%, (**c**) 40°C-RH55%, (**d**) 40 °C-RH95%, (**e**) 60 °C-RH25%, (**f**) 60 °C-RH55%, (**g**) 60 °C-RH95%, (**h**) 80 °C-RH25%, (**i**) 80 °C-RH55%, (**j**) 80 °C-RH95%.

Based on Formulas (8) and (9), the parameters A and B under different temperatures and RH can be obtained, thus the standardization stress-strain relationship of cement-based grouting material under high-geothermal environment by substituting Equations (8) or (9) into Equations (6) and (7). The full curve comparison results can be seen in Figure 15. From the comparison to the calculating results and experimental results, it can be concluded that the formula fitting curve of the model was in good agreement with the experimental curve, revealing that the proposed constitutive model can better reflect the stress-strain relationship of cement-based grouting material under high-geothermal environments.

## 4. Conclusions

In this paper, mechanical strength experiments on cement-based grouting material cured under VTC conditions were carried out to study the compressive characteristics under high-geothermal environments. The coupling effect of temperature and relative humidity on the mechanical properties of grouting material at different ages were further studied. Based on the failure characteristics and comparison to the stress-strain curves of specimens cured under SC conditions, the changing rules of typical points (peak stress and strain) of curves under different curing conditions were investigated, along with the compressive constitutive model of cement-based grouting material under high-geothermal environments. The conclusions are as follows:

(1) The compressive strength of grouting material significantly decreases with the increase of temperature. When the temperature of VTC condition is over 40 °C, it may cause a decline of 10%–40% in the mechanical properties of the grouting material, and the strength degradation is more serious under a hot-dry environment.

(2) High RH level makes a great contribution to the increase in compressive strength of grouting material, regardless of curing time. When the temperature exceeds 56.3 °C and 75 °C, the relative humidity begins to play an increasingly important role in the strength of grouting material. Moreover, the coupling effect of temperature and humidity markedly improve the grouting materials strength at early ages and decrease the degradation of long-term strength.

(3) There are similarities and differences between the compressive stress-strain curve of cement-based grouting material cured under VTC condition and SC condition. Higher temperature and lower relative humidity cause the lower relative peak stress of grouting material, and the RH effect is slightly greater than the temperature effect. As for relative peak strain, the influence of the environmental effect is to the contrary, and the temperature has a more significant influence than relative humidity on the peak strain of grouting material.

(4) According to the relationship between mechanical properties and high-geothermal environmental conditions, the calculation formula of relative stress and strain of grouting material under different environmental conditions is established. Moreover, from numerous experimental data and the complete stress-strain curves of the grouting material, a new segmental constitutive model of compressive strength in high-geothermal environment is obtained, considering both temperature and relative humidity.

## Figures and Tables

**Table 1:** Particle size distribution of cement.

Item	<5 µm (%)	<10 µm (%)	10~30 µm (%)	<30 µm (%)	Homogeneity Coefficient
Cement	9.43	31.30	41.75	73.05	1.035

**Table 2:** Particle size distribution of sand.

Item	<0.15 µm (%)	<0.3 mm (%)	<0.6 mm (%)	<1.18 mm (%)	<2.36 mm (%)	<4.75 mm (%)
Sand	3.75	27.05	49.24	70.36	96.14	100

**Table 3:** Chemical composition of Portland cement.

Item	Content (%)
SiO_2_	18.6
Al_2_O_3_	6.2
K_2_O	1.0
Na_2_O	0.2
Fe_2_O_3_	4.76
MgO	1.71
CaO	66
Material loss	1.53

**Table 4:** Experimental conditions.

Testing Item	Temperature/°C	Relative Humidity/%	Curing Mode	Curing Age/Day
Compressive strength	40, 60, 80	25, 55, 95	VTC	3, 28
	20	95	SC	3, 28

**Table 5:** Test data for peak stress-strain under different curing conditions.

Relative Humidity	Testing Item	T-20 °C	T-40 °C	T-60 °C	T-80 °C
RH25%	Peak Stress (MPa)	-	27.96	22.78	16.83
	Strain	-	2.66 × 10^−3^	2.87 × 10^−3^	3.24 × 10^−3^
RH55%	Peak Stress (MPa)	-	30.12	26.71	21.51
	Strain	-	2.25 × 10^−3^	2.45 × 10^−3^	2.87 × 10^−3^
RH95%	Peak Stress (MPa)	37.65	35.62	33.9	27.96
	Strain	1.91 × 10^−3^	2.05 × 10^−3^	2.34 × 10^−3^	2.66× 10^−3^

**Table 6:** Data collection of models and parameter estimate under different conditions.

Curing Condition	Parameter A	R2	Parameter B	R2
SC–20 °C–RH95%	0.979	0.996	3.708	0.997
VTC–40 °C–RH25%	0.749	0.99843	2.028	0.944
VTC–40 °C–RH55%	0.839	0.99584	2.19	0.9972
VTC–40 °C–RH95%	0.896	0.99611	2.916	0.989
VTC–60 °C–RH25%	0.527	0.99412	1.816	0.997
VTC–60 °C–RH55%	0.766	0.98942	1.958	0.999
VTC–60 °C–RH95%	0.831	0.999	2.358	0.980
VTC–80 °C–RH25%	0.305	0.99756	1.408	0.975
VTC–80 °C–RH55%	0.538	0.98387	1.568	0.995
VTC–80 °C–RH95%	0.755	0.99848	2.026	0.971

## References

[1] Jensen O.M., Hansen P.F. (2002). Water-entrained cement-based materials: II. Experimental observations. Cem. Concr. Res..

[2] Zong Y., Han L., Han G. (2013). Mechanical characteristics of confined grouting reinforcement for cracked rock mass. J. Min. Saf. Eng..

[3] Gu B. (2007). Countermeasures against high temperatures in a tunnel and the corresponding ventilation design—Feasibility study for the super-long Gaoligonshan tunnel. Mod. Tunn. Technol..

[4] Wang M., Tang X., Wu Q., Tong J., Dong C. (2016). Temperature Field Variation Rules of Rock and Support Structure in High Rock Temperature Tunnel. J. China Railw. Soc..

[5] Byle M.J. (1995). Verification of Geotechnical Grouting.

[6] Skjølsvold O., Justnes H. (2015). TIGHT-Prøving av Injiseringssementer, Laboratorieprøving.

[7] Li H., Yang K., Guan X. (2019). Properties of sulfoaluminate cement-based grouting materials modified with LiAl-layered double hydroxides in the presence of PCE superplasticizer. Constr. Build. Mater..

[8] Li S., Zhang J., Li Z., Gao Y., Qi Y., Li H., Zhang Q. (2019). Investigation and practical application of a new cementitious anti-washout grouting material. Constr. Build. Mater..

[9] Won J.-P., Hwang U.-J., Kim C.-K., Lee S.-J. (2013). Mechanical performance of shotcrete made with a high-strength cement-based mineral accelerator. Constr. Build. Mater..

[10] Wang M., Hu Y., Wang Q., Tian H., Liu D. (2019). A study on strength characteristics of concrete under variable temperature curing conditions in ultra-high geothermal tunnels. Constr. Build. Mater..

[11] Widmann R. (1996). International Society for Rock Mechanics Commission on Rock Grouting. International Journal of Rock Mechanics and Mining Sciences & Geomechanics Abstracts.

[12] Mirza J., Saleh K., Langevin M.-A., Mirza S., Bhutta M.A.R., Tahir M.M. (2013). Properties of microfine cement grouts at 4 C, 10 C and 20 C. Constr. Build. Mater..

[13] Elkhadiri I., Palacios M., Puertas F. (2009). Effect of curing temperature on cement hydration. Ceram Silik.

[14] Holt E., Leivo M. (2004). Cracking risks associated with early age shrinkage. Cem. Concr. Compos..

[15] Bohloli B., Skjolsvold O., Justnes H., Olsson R., Grov E., Aarset A. (2019). Cements for tunnel grouting—Rheology and flow properties tested at different temperatures. Tunn. Undergr. Space Technol..

[16] Chen B., Li C., Chen L. (2009). Experimental study of mechanical properties of normal-strength concrete exposed to high temperatures at an early age. Fire Saf. J..

[17] Wang L., Yuan G.-L., Zhou L.-X. (2011). Study on the mechanical property of early age concrete columns after high temperature. Concrete.

[18] Li Q., Liu L., Huang Z., Yuan G. (2017). Residual compressive strength of cement-based grouting material with early ages after fire. Constr. Build. Mater..

[19] Li Q., Liu L., Huang Z., Yuan G. (2018). Degradation of the elastic modulus of cement-based grouting material with early ages after fire. Constr. Build. Mater..

[20] Hu Y., Wang M., Wang Q., Liu D., Tong J. (2019). Field test of thermal environment and thermal adaptation of workers in high geothermal tunnel. Build. Environ..

[21] Mingnian W., Yunpeng H.U., Jianjun T., Qiling W., Yicheng W., Congyu D. (2019). Experimental study on shear mechanical properties and thermal damage model of shotcrete-rock interfaces under variable high temperatures. Chin. J. Rock Mech. Eng..

[22] Cui S., Xu D., Liu P., Ye Y. (2017). Exploratory Study on Improving Bond Strength of Shotcrete in Hot and Dry Environments of High Geothermal Tunnels. KSCE J. Civ. Eng..

[23] Cui S.G., Liu P., Su J., Cui E.Q., Guo C., Zhu B. (2018). Experimental study on mechanical and microstructural properties of cement-based paste for shotcrete use in high-temperature geothermal environment. Constr. Build. Mater..

[24] (2007). Common Portland Cement (GB175-2007).

[25] Goy L., Fabre D., Menard G. (1996). Modelling of rock temperatures for deep alpine tunnel projects. Rock Mech. Rock Eng..

[26] Wilhelm J., Rybach L. (2003). The geothermal potential of Swiss Alpine tunnels. Geothermics.

[27] Koltzer N., Scheck-Wenderoth M., Bott J., Cacace M., Frick M., Sass I., Fritsche J.-G., Bär K. (2019). The Effects of Regional Fluid Flow on Deep Temperatures (Hesse, Germany). Energies.

[28] Liu X., Yao Z., Xue W., Li X. (2019). Development, Performance, and Microscopic Analysis of New Anchorage Agent with Heat Resistance, High Strength, and Full Length. Adv. Mater. Sci. Eng..

[29] Abdus S., Cheng X., Huang W., Ahmed A., Hu R. (2019). Bearing failure and influence factors analysis of metal-to-composite bolted joints at high temperature. J. Braz. Soc. Mech. Sci. Eng..

[30] Yuan P. (2014). Discussion on key technologies for construction of water diversion tunnel under super-high ground temperature. Water Resour. Hydropower Eng. (Chin.).

[31] Lan S.R., Guo Z.H. (1999). Biaxial compression behavior of concrete under repeated loading. J. Mater. Civ. Eng..

[32] Zhenhai G. (1997). Strength and Deformation of Concrete—Experimental Basis and Constitutive Relationship.

